# Effect of Adjuvant Paclitaxel and Carboplatin on Survival in Women With Triple-Negative Breast Cancer: A Phase 3 Randomized Clinical Trial

**DOI:** 10.1001/jamaoncol.2020.2965

**Published:** 2020-08-13

**Authors:** Ke-Da Yu, Fu-Gui Ye, Min He, Lei Fan, Ding Ma, Miao Mo, Jiong Wu, Guang-Yu Liu, Gen-Hong Di, Xiao-Hua Zeng, Ping-Qing He, Ke-Jin Wu, Yi-Feng Hou, Jie Wang, Cheng Wang, Zhi-Gang Zhuang, Chuan-Gui Song, Xiao-Yan Lin, Angela Toss, Francesco Ricci, Zhen-Zhou Shen, Zhi-Ming Shao

**Affiliations:** 1 Department of Breast Surgery, Fudan University Shanghai Cancer Center, Shanghai, China; 2 Department of Cancer Prevention & Clinical Statistics Center, Fudan University Shanghai Cancer Center, Shanghai, China; 3 Breast Center, Chongqing Cancer Hospital, Chongqing University, Chongqing, China; 4 Department of Breast Surgery, Shanghai Sixth People’s Hospital, Shanghai Jiao Tong University, Shanghai, China; 5 Department of Breast Surgery, Obstetrics and Gynecology Hospital of Fudan University, Shanghai, China.; 6 Department of Breast Surgery, The International Peace Maternity & Child Health Hospital of China Welfare Institute, Shanghai Jiao Tong University, Shanghai, China; 7 Department of Breast Surgery, Shanghai Ninth People’s Hospital Huangpu Branch, Shanghai Jiao Tong University, Shanghai, China.; 8 Department of Breast Surgery, Shanghai First Maternity and Infant Hospital, Shanghai Tongji University, Shanghai, China; 9 Department of Breast Surgery, Fujian Medical University Union Hospital, Fuzhou, China; 10 Department of Breast Surgery, Tongji University School of Medicine Yangpu Hospital, Shanghai, China; 11 Department of Oncology and Hematology, University Hospital of Modena, Modena, Italy; 12 Department of Drug Development and Innovation, Institute Curie, Paris & Saint-Cloud, France; 13 Key Laboratory of Breast Cancer in Shanghai, Shanghai, China

## Abstract

**Importance:**

The value of platinum-based adjuvant chemotherapy in patients with triple-negative breast cancer (TNBC) remains controversial, as does whether *BRCA1* and *BRCA2* (*BRCA1/2*) germline variants are associated with platinum treatment sensitivity.

**Objective:**

To compare 6 cycles of paclitaxel plus carboplatin (PCb) with a standard-dose regimen of 3 cycles of cyclophosphamide, epirubicin, and fluorouracil followed by 3 cycles of docetaxel (CEF-T).

**Design, Setting, and Participants:**

This phase 3 randomized clinical trial was conducted at 9 cancer centers and hospitals in China. Between July 1, 2011, and April 30, 2016, women aged 18 to 70 years with operable TNBC after definitive surgery (having pathologically confirmed regional node-positive disease or node-negative disease with tumor diameter >10 mm) were screened and enrolled. Exclusion criteria included having metastatic or locally advanced disease, having non-TNBC, or receiving preoperative anticancer therapy. Data were analyzed from December 1, 2019, to January 31, 2020, from the intent-to-treat population as prespecified in the protocol.

**Interventions:**

Participants were randomized to receive PCb (paclitaxel 80 mg/m^2^ and carboplatin [area under the curve = 2] on days 1, 8, and 15 every 28 days for 6 cycles) or CEF-T (cyclophosphamide 500 mg/m^2^, epirubicin 100 mg/m^2^, and fluorouracil 500 mg/m^2^ every 3 weeks for 3 cycles followed by docetaxel 100 mg/m^2^ every 3 weeks for 3 cycles).

**Main Outcomes and Measures:**

The primary end point was disease-free survival (DFS). Secondary end points included overall survival, distant DFS, relapse-free survival, DFS in patients with germline variants in *BRCA1/2* or homologous recombination repair (HRR)–related genes, and toxicity.

**Results:**

A total of 647 patients (mean [SD] age, 51 [44-57] years) with operable TNBC were randomized to receive CEF-T (n = 322) or PCb (n = 325). At a median follow-up of 62 months, DFS time was longer in those assigned to PCb compared with CEF-T (5-year DFS, 86.5% vs 80.3%, hazard ratio [HR] = 0.65; 95% CI, 0.44-0.96; *P* = .03). Similar outcomes were observed for distant DFS and relapse-free survival. There was no statistically significant difference in overall survival between the groups (HR = 0.71; 95% CI, 0.42-1.22, *P* = .22). In the exploratory and hypothesis-generating subgroup analyses of PCb vs CEF-T, the HR for DFS was 0.44 (95% CI, 0.15-1.31; *P* = .14) in patients with the *BRCA1/2* variant and 0.39 (95% CI, 0.15-0.99; *P* = .04) in those with the HRR variant. Safety data were consistent with the known safety profiles of relevant drugs.

**Conclusions and Relevance:**

These findings suggest that a paclitaxel-plus-carboplatin regimen is an effective alternative adjuvant chemotherapy choice for patients with operable TNBC. In the era of molecular classification, subsets of TNBC sensitive to PCb should be further investigated.

**Trial Registration:**

ClinicalTrials.gov Identifier: NCT01216111

## Key Points

**Question:**

Does a paclitaxel-plus-carboplatin (PCb) as adjuvant treatment in women with operable triple-negative breast cancer offer superior benefit compared with a standard-dose CEF-T regimen (cyclophosphamide, epirubicin, and fluorouracil followed by docetaxel)?

**Findings:**

In this randomized phase 3 clinical trial conducted at 9 cancer centers and hospitals in China and including 647 patients, after a median follow-up of 62 months, 5-year disease-free survival rate was statistically significantly higher in the PCb group compared with the CEF-T group.

**Meaning:**

Results of this study suggest that a paclitaxel-plus-carboplatin regimen may be an alternative adjuvant chemotherapy choice for patients with operable triple-negative breast cancer.

## Teaser

This randomized phase 3 clinical trial evaluates the effect of a carboplatin-plus-paclitaxel regimen on survival, compared with a standard-dose regimen of cyclophosphamide, epirubicin, and fluorouracil followed by docetaxel, in women with operable triple-negative breast cancer in China.

## Introduction

Approximately 15% to 20% of breast cancers are classified as triple-negative breast cancer (TNBC), which is characterized by a lack of estrogen receptor and progesterone receptor expression and no *ERBB2* gene amplification.^1^ Compared with other subtypes, TNBC tends to have a higher histologic grade, increased aggressiveness, and more risk of local recurrence and visceral metastasis.^2^ The reported prevalence of *BRCA1* and *BRCA2* (*BRCA1/2*) variants in unselected patients with TNBC is 11.2%.^3^ Both *BRCA*-associated breast cancer and sporadic TNBC exhibit characteristics consistent with abnormal DNA repair and genome-wide instability, which supports the use of DNA-damaging compounds, such as platinum derivatives.^2^

Platinum agents are cytotoxic DNA-damaging compounds that cause DNA strand breaks and consequently lead to apoptosis; this unique mechanism of action renders these drugs particularly active in cancer cells with DNA repair deficiency, such as those harboring deleterious variants in *BRCA* genes.^4^ On the basis of this biologic rationale, several studies have investigated the possible role of platinum agents in the treatment of patients with TNBC in both metastatic and neoadjuvant settings. For example, the results from the Triple Negative Trial^5^ suggested that carboplatin performed similarly to docetaxel as first-line therapy in patients with unselected metastatic TNBC but that the former was more effective in patients carrying *BRCA* variants. The CBCSG006 trial^6^ reported superior efficacy for the cisplatin-plus-gemcitabine regimen vs the paclitaxel-plus-gemcitabine regimen as first-line chemotherapy for patients with metastatic TNBC. In the neoadjuvant setting, several randomized controlled trials have found that the addition of carboplatin on the backbone of a taxane with or without an anthracycline increased the proportion of pathological complete response.^7,8^

The value of platinum-based chemotherapy in the adjuvant setting in TNBC patients remains controversial. We initiated the present PATTERN (adjuvant Platinum and Taxane in Triple-Negative Breast Cancer) trial to investigate whether a paclitaxel-plus-carboplatin regimen as adjuvant treatment in TNBC patients would offer superior benefit compared with a standard regimen of anthracycline and docetaxel.

## Methods

### Study Design and Participants

The PATTERN trial is a randomized, open-label, multicenter, phase 3 clinical trial performed in 9 cancer centers and hospitals in China (eTable 1 in Supplement 2) designed on the premise of demonstrating the effects of a carboplatin-containing regimen. The independent institutional review boards of the participating centers approved the study protocol (Supplement 1). This trial followed the Consolidated Standards of Reporting Trials (CONSORT) reporting guideline. We performed the study according to the International Conference on Harmonisation Good Clinical Practice guidelines and ethical principles of the Declaration of Helsinki.^9^ All patients provided written informed consent.

Patients were screened between July 1, 2011, and April 30, 2016. Women aged 18 to 70 years with operable, primary invasive TNBC were eligible for enrollment following definitive surgery. Patients were eligible if they had pathologically confirmed regional node-positive disease or node-negative disease with primary tumor diameter >10 mm. Estrogen receptor, progesterone receptor, and *ERBB2* statuses were identified locally at each participating center based on immunohistochemical analysis of tumor sections. The immunohistochemical cutoff for estrogen receptor/progesterone receptor–negative status was less than 1% staining in nuclei according to the American Society of Clinical Oncology/College Of American Pathologists test guideline.^10^ *ERBB2*-negative status was a score of 0 or 1 by immunohistochemical analysis or the absence of *ERBB2 *amplification by fluorescence in situ hybridization with an immunohistochemistry score of 2 according to the American Society of Clinical Oncology/College Of American Pathologists guideline.^11^ Eastern Cooperative Oncology Group performance status was required to be less than 2, and the following criteria for adequate organ function needed to be met: adequate hematologic function and hepatic and renal function and normal cardiac function.

Patients were ineligible if they had metastatic or locally advanced disease, had non-TNBC, or received preoperative anticancer therapy (including chemotherapy and radiotherapy). Full inclusion and exclusion criteria can be found in the study protocol (Supplement 1).

We hypothesized that carriers with deleterious *gBRCA1/2* variants might obtain more survival benefits from carboplatin-containing chemotherapy than with the traditional regimen.^12^ For all participants, a germline variant test of *BRCA1/2* was recommended but not mandatory; variant status was determined using blood samples obtained before chemotherapy. As the *BRCA1/2* test requires time and we could not obtain variant information before chemotherapy was initiated, randomization could not be stratified according to *gBRCA1/2* status. All coding regions and exon-intron boundaries of the *BRCA1/2* genes were screened. Genetic testing was conducted in the central laboratory of Fudan University Shanghai Cancer Center (FUSCC).^13^ The detailed sequencing procedure and interpretation of the variants are described in the eMethods in Supplement 2. All variants considered pathogenic or likely pathogenic were validated via Sanger sequencing.

With the development of this trial, several novel reports have shown that *BRCA1/2* variant is not the only surrogate of homologous recombination deficiency (HRD), which is probably an indicator of platinum salt sensitivity,^14^ and a few assays for HRD status have been reported.^15,16^ Thus, we examined a set of genes involved in homologous recombination repair (HRR) using DNA samples from peripheral blood in a 2-step process. First, we identified candidate HRR genes with germline variants in the Chinese population. By testing 405 patients with TNBC (samples obtained between 2005 and 2010 in FUSCC) using whole exome sequencing, as described elsewhere,^17^ we found germline variants in 12 HRR-related genes: *ATM*, *ATR*, *BARD1*, *BRCA1*, *BRCA2*, *BRIP1*, *CHEK2*, *FANCM*, *PALB2*, *RAD51C*, *RAD51D*, and *RECQL*. Second, we assessed germline variants in these 12 HRR-related genes in the trial population. The details of the methodology are provided in the eMethods in Supplement 2.

### Randomization and Masking

We randomly assigned eligible patients (1:1) to receive either paclitaxel and carboplatin (PCb) or cyclophosphamide, epirubicin, and fluorouracil followed by docetaxel (CEF-T). Randomization was performed via an interactive web-response system.^6^ Investigators sent the random assignment forms by fax to the research coordination office in FUSCC. The study coordinator sent the allocated treatment group to the investigator by fax after checking the inclusion and exclusion criteria. Randomization was stratified according to pathological node status (negative vs positive), age (younger than 50 years vs 50 years and older), and pathological tumor size (pT1 vs pT2-3). Because this was an open-label study, the patients, investigators, and study team were not masked to the treatment group.

### Procedures

The participants’ baseline characteristics were recorded at randomization. Patients were randomly assigned to receive PCb, ie, paclitaxel 80 mg/m^2^ and carboplatin (area under the curve = 2) on days 1, 8, and 15 every 28 days for 6 cycles; or CEF-T, ie, fluorouracil 500 mg/m^2^, epirubicin 100 mg/m^2^, and cyclophosphamide 500 mg/m^2^ intravenously on day 1 every 3 weeks for 3 cycles followed by docetaxel 100 mg/m^2^ intravenously on day 1 every 3 weeks for 3 cycles. Chemotherapy should be initially administered within 8 weeks after initial breast cancer surgery. Concurrent or extended treatments of adjuvant capecitabine as well as other chemotherapeutic regimens were forbidden. Timing of adjuvant radiotherapy and the follow-up schedule are described in the eMethods in Supplement 2.

### Outcomes

The primary end point was disease-free survival (DFS). The DFS events included noninvasive and invasive breast cancer recurrences (local, regional, or distant), second primary noninvasive and invasive breast and cancers other than basal/squamous-cell carcinoma of the skin and carcinoma in situ of the cervix, and death from any cause. Secondary end points included the following: distant disease-free survival (DDFS), relapse-free survival (RFS), overall survival (OS), and toxicity. Detailed definitions of survival outcomes are provided in the eMethods in Supplement 2. Another prespecified secondary end point was DFS in *gBRCA1* variant carriers. An exploratory analysis of the interaction between carboplatin-containing chemotherapy and HRR-related gene variant was amended in 2017. Toxicity was graded according to National Cancer Institute Common Toxicity Criteria version 4.0 and was assessed in patients who received at least 1 cycle of chemotherapy.^18^

### Statistical Analysis

Data were analyzed from December 1, 2019, to January 31, 2020. The study was designed to detect an absolute 7% improvement in the 5-year DFS rate for the PCb group (assumed 89%) compared with the CEF-T group (assumed 82%), with a corresponding hazard ratio (HR) of 0.60. Under these assumptions, a 2-sided log-rank test with 80% power required 614 patients (307 in the CEF-T group and 307 in the PCb group) to show a 5% level of significance, with 112 DFS events expected after a minimum 5-year follow-up time. Considering 5% loss to follow-up, 645 randomized patients were required. No interim analysis was planned for DFS. In a superiority trial, a 1-sided *P* value (at a significance level of .025) should be used for decision-making in analyses. A 1-sided *P* value at a significance level of .025 corresponded to a 2-sided *P* value at a significance level of .05. We determined 2-sided *P* values at a significance level of .05 according to usual practice.

The intention-to-treat principle was used for the primary analysis, which was performed on all randomly assigned patients with follow-up. For continuous and categorical factors, the Wilcoxon rank-sum test and the χ^2^ test (or Fisher exact test when necessary) were used to evaluate differences between the 2 groups. The Kaplan-Meier method was used to estimate the distributions of survival outcomes. Comparisons in survival rates between the treatment groups were assessed by the stratified log-rank test. Hazard ratios and 95% CIs were obtained using the stratified Cox proportional hazards model. The Cox model was also used to control intergroup confounding prognostic variables. In addition, we performed a test for the interaction between treatment and clinicopathological factors. The stratification factors at randomization were used in stratified analyses unless otherwise stated. All analyses were conducted using the R programming language and environment, version 3.5.0 (R Project for Statistical Computing) and STATA statistical software, version 16.0 (StataCorp LLC).

## Results

**Figure 1.**
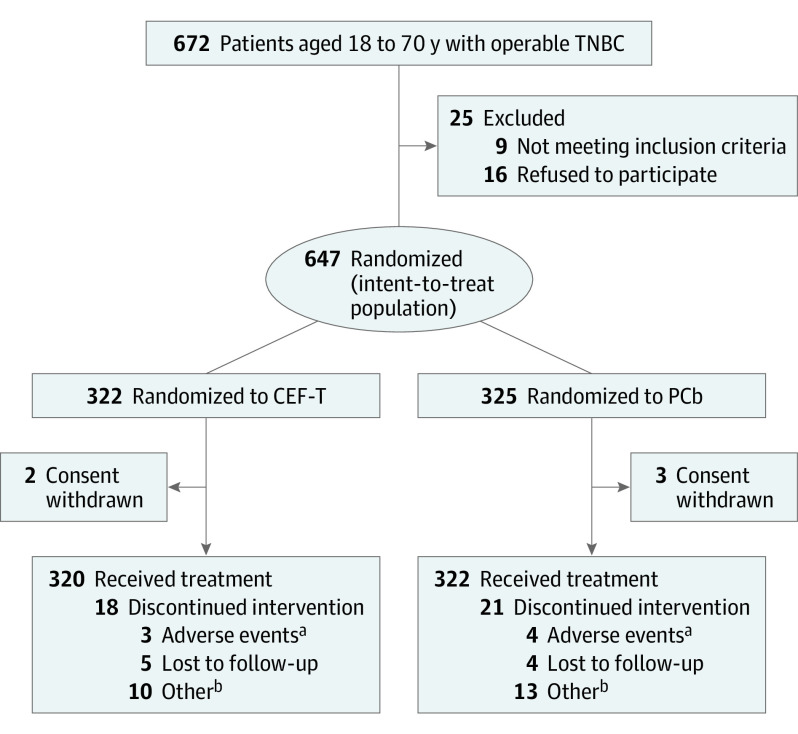
Patient Flow Diagram CEF-T indicates fluorouracil, epirubicin, and cyclophosphamide followed by docetaxel; PCb, paclitaxel and carboplatin; and TNBC, triple-negative breast cancer. ^a^Adverse events indicate grades 3 and 4. ^b^Other reasons except for adverse events.

Between July 1, 2011, and April 30, 2016, 672 women with TNBC were screened at 9 cancer centers and hospitals in China, and 647 patients were enrolled and randomly assigned to 2 treatment groups: 322 in the CEF-T group and 325 in the PCb group (Figure 1). Baseline characteristics were well balanced between the groups (Table 1). The median age was 51 years (interquartile range, 44-57 years) at the time of study entry. Most enrolled patients had early-stage TNBC (74% were node-negative; median number of positive nodes, 2 [interquartile range, 1-4]). Chemotherapy was completed by 305 patients (94.7%) in the CEF-T group and by 302 patients (92.9%) in the PCb group. Dose reductions were needed for 30 patients (9.3%) in the CEF-T group and 28 patients (8.6%) in the PCb group.

**Table 1.** Patient Characteristics by Treatment Group

Characteristic	No. (%)		
	Total (N = 647)	CEF-T (n = 322)	PCb (n = 325)
Age, median (IQR), y	51 (44-57)	50 (44-57)	51 (44-57)
Pathologic tumor size			
pT1	351 (54.2)	173 (53.7)	178 (54.8)
pT2-pT3	296 (45.8)	149 (46.3)	147 (45.2)
Node status			
Negative	481 (74.3)	244 (75.8)	237 (72.9)
Positive	166 (25.7)	78 (24.2)	88 (27.1)
Histological grade			
I-II	177 (27.4)	88 (27.3)	89 (27.4)
III	470 (72.6)	234 (72.7)	236 (72.6)
Ki67 proliferation index (%)			
≤14	80 (12.4)	40 (12.4)	40 (12.3)
>14	567 (87.6)	282 (87.6)	285 (87.7)
Surgery			
BCS	203 (31.4)	99 (30.7)	104 (32.0)
Mastectomy	444 (68.6)	223 (69.3)	221 (68.0)
Adjuvant radiation			
Yes	296 (45.7)	144 (44.7)	152 (46.8)
No	351 (54.3)	178 (55.3)	173 (53.2)
*BRCA1/2* genes			
Deleterious variant^a^	66 (10.2)	32 (9.9)	34 (10.5)
No deleterious variant	472 (73.0)	237 (73.6)	235 (72.3)
Unknown	109 (16.8)	53 (16.5)	56 (17.2)
HRR-related genes			
Deleterious variant	120 (18.5)	61 (18.9)	59 (18.1)
No deleterious variant	401 (62.0)	199 (61.8)	202 (62.2)
Unknown	126 (19.5)	62 (19.3)	64 (19.7)

Abbreviations: BCS, breast conservative surgery; CEF-T, fluorouracil, epirubicin, and cyclophosphamide followed by docetaxel; HRR, homologous recombination repair; IQR, interquartile range; PCb, paclitaxel and carboplatin.

^a^ When patients with unknown results were excluded, the deleterious variant rates in *BRCA1/2* were 12.3%, 11.9%, and 12.6% for the full population, the CEF-T group, and the PCb group, respectively.

**Figure 2.**
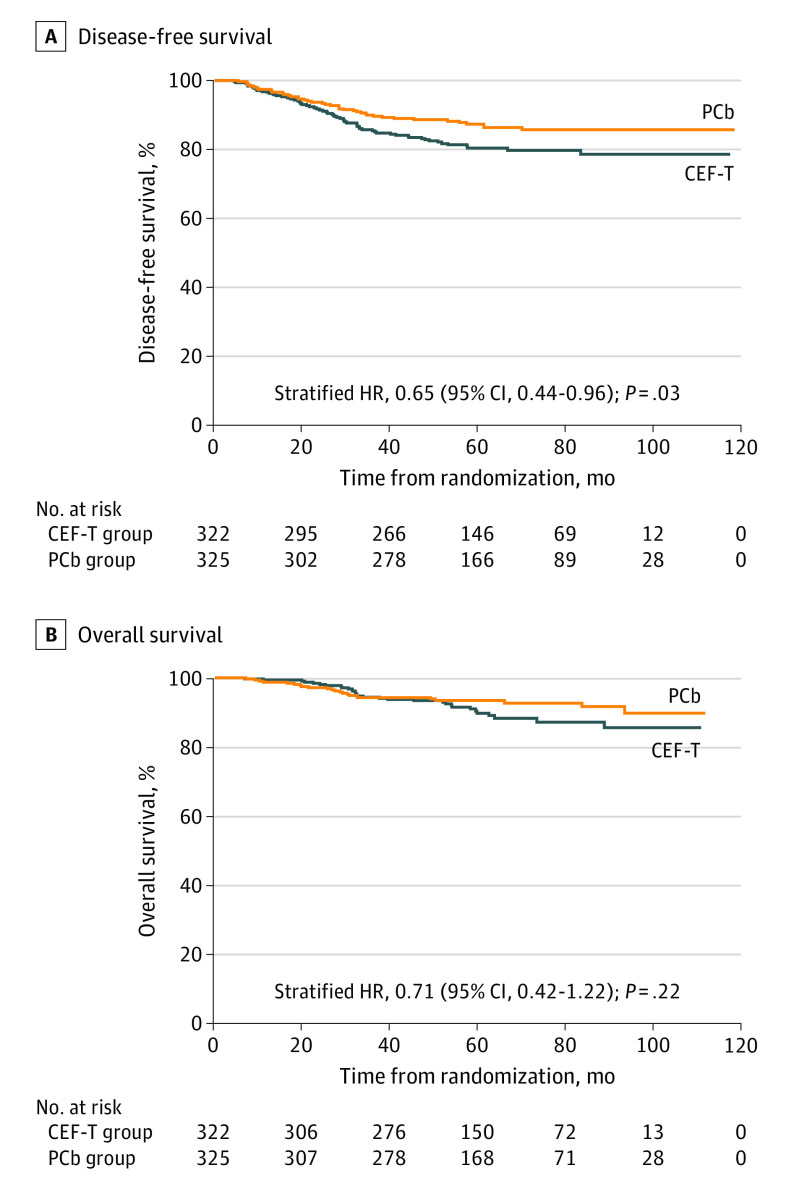
Disease-Free Survival and Overall Survival Kaplan-Meier plots show disease-free survival (A) and overall survival (B) for the entire population. CEF-T indicates fluorouracil, epirubicin, and cyclophosphamide followed by docetaxel; HR, hazard ratio; and PCb, paclitaxel and carboplatin.

At a median follow-up of 62 months, 104 of 647 randomized patients (16.1%) experienced DFS events, as summarized in Table 2. The Kaplan-Meier curves for DFS are depicted in Figure 2A. The absolute benefit of the 5-year DFS rate was 6.4% for the PCb group, which was greater than that of the CEF-T group (86.5% vs 80.3%), with a statistically significant difference (42 events among 325 patients in the PCb group vs 62 events among 322 patients in the CEF-T group; HR, 0.65; 95% CI, 0.44-0.96; stratified log-rank *P* = .03).

**Table 2.** First Disease-Free Survival Event by Treatment

Disease-free survival event	No. (%)	
	CEF-T (n = 322)	PCb (n = 325)
Local and regional recurrence	10 (3.1)	4 (1.2)
Contralateral breast tumor	8 (2.5)	9 (2.8)
Distant metastasis	33 (10.2)	20 (6.2)
Second primary malignancy	8 (2.5)	7 (2.2)
Death	3 (0.9)	2 (0.6)
Total	62 (19.3)	42 (12.9)

Abbreviations: CEF-T, fluorouracil, epirubicin, and cyclophosphamide followed by docetaxel; PCb, paclitaxel and carboplatin.

The Kaplan-Meier curve for OS is shown in Figure 2B, and the Kaplan-Meier curves for RFS and DDFS are in eFigure 1 in Supplement 2. Although there was no significant difference in 5-year OS between the 2 treatment groups (23 events among 325 patients in the PCb group vs 31 events among 322 patients in the CEF-T group; OS, 93.4% vs 89.8%; HR, 0.71; 95% CI, 0.42-1.22; *P* = .22), adequate efficacy assessment of PCb on OS will require long-term follow-up and more events. RFS at 5 years was higher in the PCb group than in the CEF-T group (26 events among 325 patients vs 46 events among 322 patients; RFS, 91.2% vs 84.4%, HR, 0.54; 95% CI, 0.34-0.88; *P* = .01), and similar results were observed for DDFS (22 events among 325 patients vs 36 events among 322 patients; DDFS, 92.6% vs 87.9%; HR, 0.59; 95% CI, 0.35-0.999; *P* = .05).

**Figure 3.**
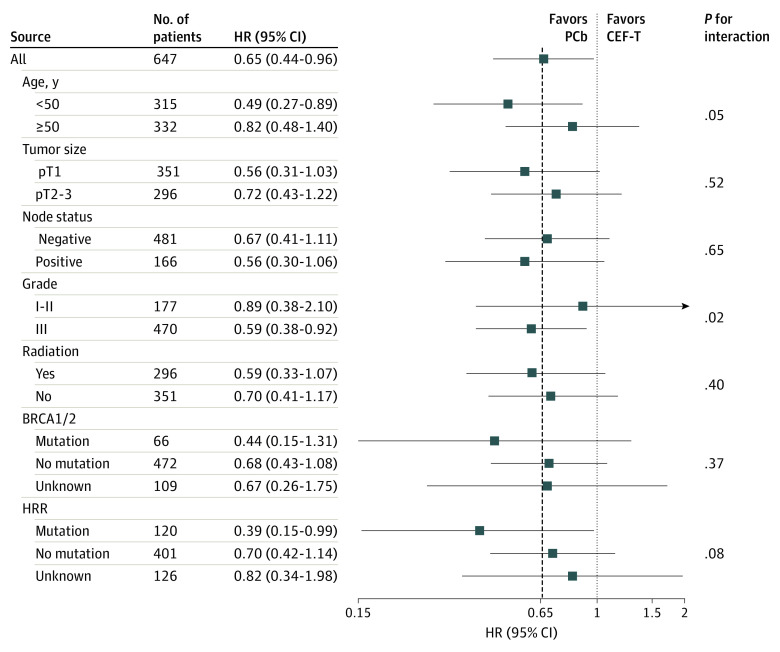
Forest Plots of Exploratory Subgroup Analysis of Disease-Free Survival CEF-T indicates fluorouracil, epirubicin, and cyclophosphamide followed by docetaxel; HR, hazard ratio; HRR, homologous recombination repair; and PCb, paclitaxel and carboplatin.

According to the exploratory subgroup analyses of DFS, patients with younger age or high-grade tumor appeared to have benefited more from PCb (Figure 3). We focused particularly on the subgroup analysis of 538 patients with known *gBRCA1/2* status. The HR for the effect of PCb vs CEF-T on DFS according to *gBRCA1/2* status was 0.44 (95% CI, 0.15-0.31; *P* = .14) for patients with this variant and 0.68 (95% CI, 0.43-1.08; *P* = .10) for those without this variant, with an interaction *P* value of 0.37, indicating that this difference could be attributed to chance given the small number of participants with *BRCA* variants (n = 66). The effects of the interaction of *BRCA1/2* status and chemotherapy regimens on DFS are illustrated in eFigure 2A in Supplement 2.

We further assessed HRD status by testing germline variants in HRR-related genes. First, we analyzed the spectrum of germline variants in the Chinese TNBC population and found 22 genes with germline variants. Among them, 12 genes are related to HRR; the variant rate was 20.2% in TNBC cases (eTable 2 in Supplement 2). Next, we assessed the variants of 12 HRR genes in 472 non-*BRCA1/2* carriers of the trial population. Among them, 17 patients were not tested because of the unavailability of DNA samples, and 54 showed deleterious variants in HRR genes. Overall, there were 120 carriers of deleterious HRR variants (66 with *BRCA1/2* variants and 54 with non-*BRCA1/2* variants) and 401 noncarriers. For the 54 carriers with HRR other than a *BRCA1/2* variant, the DFS HR for PCb vs CEF-T was 0.12 (95% CI, 0.02-1.28; *P* = .08) owing to a small sample size and rare events. In the combination analysis, HRR variant carriers displayed a significantly better DFS with the PCb regimen compared with the CEF-T regimen (88.4% vs 76.3%; HR, 0.39; 95% CI, 0.15-0.99; *P* = .04). In contrast, the DFS difference between PCb and CEF-T was insignificant among noncarriers (85.9% vs 81.2%; HR, 0.70; 95% CI, 0.42-1.14; *P* = .15), with a borderline *P* value of 0.08 for interaction. The Kaplan-Meier plots of interactions between HRR status and chemotherapy regimens on DFS are shown in eFigure 2B in Supplement 2.

Treatment-related grades 3 to 4 adverse events are listed in eTable 3 in Supplement 2. Both treatments were generally well tolerated, and treatment-related deaths or life-threatening events were not observed. As expected, hematologic toxicity was predominant with both regimens. Grade 3 or 4 febrile neutropenia occurred more often in patients allocated to the CEF-T group, whereas peripheral neuropathy, anemia, and thrombocytopenia were more common in the PCb group.

## Discussion

The PATTERN trial was designed to determine whether PCb is superior to CEF-T in the adjuvant settings of TNBC, and the results indicated the greater benefit of the platinum-based regimen compared with the standard anthracycline/taxane regimen. To our knowledge, this is the first randomized trial comparing adjuvant PCb with CEF-T in patients with TNBC.

Triple-negative breast cancer is often associated with a dysfunctional *BRCA* pathway with deficient mismatch repair and genomic instability^19^; correspondingly, platinum-containing regimens have been proven to be effective in *gBRCA1/2* variant carriers.^12^ A meta-analysis that included the pivotal trials GeparSixto^8^ and CALGB 40603^7^ confirmed the activity of adding platinum for treatment of patients with the *gBRCA* variant compared with non-*BRCA1/2* carriers in the neoadjuvant setting.^20^ Moreover, in the neoadjuvant GeparSixto study, adding carboplatin resulted in significantly better DFS in patients with TNBC.^14^ In the adjuvant setting, however, such evidence is lacking. We demonstrated for the first time to our knowledge that the carboplatin-containing regimen is superior to the anthracycline/taxane regimen for early-stage TNBC. The survival results from GeparSixto are consistent with our findings. It is worth noting that the absolute DFS benefit in GeparSixto of 10% was higher than that in our study (6.2%). The reason might be that our participants had a lower tumor burden, namely, 54% were pT1 tumors and 74% were node-negative tumors, and correspondingly the degree of benefit was relatively small. The strength of our study is that DFS was designed as the primary end point, with sufficient sample size and adequate statistical power.

Although the observed differences in DFS were significant in the entire population with unselected TNBC, a clinical profile of patients sensitive to the PCb regimen might show similar characteristics, such as younger age and higher tumor grade, according to the exploratory subgroup analyses. The exploratory analysis also indicated a strong association with treatment benefit from a carboplatin-containing regimen in patients with TNBC with defects in homologous recombination. Initially, we hypothesized that the *BRCA1/2* variant is an indicator for carboplatin treatment, as *BRCA1/2* is a clinically validated biomarker that is associated with a greater response in patients treated with carboplatin than in those treated with other therapeutic drugs, but we failed to prove this hypothesis in our study. Potential explanations are as follows. First, the sample size of *BRCA1/2* variant carriers was small, and the statistics for difference comparison were underpowered. Second, even among *gBRCA1/2*-negative patients, some might be sensitive to carboplatin because of HRD due to HRR variation rather than to *BRCA1/2* variants, and thus non-*BRCA1/2* HRR variant carriers would dilute and affect the results. Therefore, we further stratified patients according to HRR variant and demonstrated that patients carrying HRR variants might benefit more from PCb than from CEF-T. However, our findings from exploratory subgroup analysis are hypothesis-generating, and further prospective randomized trials are required to confirm our results.

### Limitations

There are some limitations in our study. First, CEF-T, a preferred regimen when the trial was first designed, is no longer a primary recommendation in the National Comprehensive Cancer Network guidelines. According to updated data from ECOG 1199,^21^ epirubicin and cyclophosphamide followed by weekly paclitaxel (EC-wP) might be the optimal choice for TNBC. However, we lack a head-to-head comparison between CEF-T and EC-wP, and we must point out that our trial predominantly enrolled patients with early-stage TNBC (74% were node-negative, and the median number of positive nodes was 2 [interquartile range, 1-4]). In such patients with a lower tumor burden, whether an EC-wP regimen is the optimal choice remains inconclusive. Second, the current study was limited to Chinese patients, and validation trials for ethnic extrapolation are warranted. Third, the current analysis regarding HRR variation or *BRCA1/2* subgroups was underpowered because of the relatively small sample size, and some germline variants might not have been included in the set of 12 HRR-related genes. Our findings suggest that deleterious variants in *BRCA1/2* occur in 12% of TNBC cases and that variants in other non-*BRCA1/2* genes occur in approximately 8% to 10% of cases. A standard approach for genetic testing in TNBC is needed for risk assessment as well as treatment guidance in the era of precision medicine. Finally, genetic and epigenetic inactivation of homologous recombination might also lead to HRD, and the development of different surrogates of HRD will be key to expanding the therapeutic utility of HRD-targeting agents across a broad spectrum of tumor types.

## Conclusions

In conclusion, this randomized clinical trial found that compared with the conventional anthracycline and docetaxel regimen, the paclitaxel-plus-carboplatin regimen may be an alternative adjuvant chemotherapy strategy for patients with operable TNBC. However, the results should be considered with caution, as high-level evidence is still lacking to make platinum-based chemotherapy the new standard of care. Moreover, identifying predictive biomarkers is imperative for the selection of appropriate patients for platinum-based regimens in the adjuvant setting.

## Notes

Supplement 1. **Trial Protocol**

Supplement 2. **eMethods.** Protocol and Participating Hospitals **eTable 1.** Recruitment of Patients by Hospital and Institution **eTable 2.** Genes with Germline Mutation in 405 TNBC Using Whole Exome Sequencing Assay **eTable 3.** Grade 3 to 4 Treatment-Related Adverse Events **eFigure 1.** Kaplan-Meier Plots of (A) Distant Disease-Free Survival and (B) Relapse-Free Survival **eFigure 2.** Kaplan-Meier Plots of Disease-Free Survival According to BRCA1 Germline Mutation (A) and HRR Germline Mutation Status (B)

Supplement 3. **Data Sharing Statement**
