# Supplementary material for: Effect of Adjuvant Paclitaxel and Carboplatin on Survival in Women With Triple-Negative Breast Cancer: A Phase 3 Randomized Clinical Trial
Source: JAMA Oncol. 2020 Aug 13;6(9):1–8. doi: 10.1001/jamaoncol.2020.2965 (PMC7426881; doi:10.1001/jamaoncol.2020.2965)
Supplement: Supplement 1. — Trial Protocol [file jamaoncol-e202965-s001.pdf]

Adjuvant Platinum and Taxane in Triple-negative breast cancer (PATTERN): A Randomized, Controlled, Phase III study

Study protocol

Study institute: Fudan University Shanghai Cancer Center

Major study investigator: Zhi-Ming Shao, MD.

ClinicalTrials.gov Identifier: NCT01216111

Tables of Contents

1. Study protocol summary 4
2. Experiment background 5
3. Experiment objective 6
4. Study design 6
5. Participants enrollment 6
   - 5.1 Inclusion criteria: 7
   - 5.2 Exclusion criteria 8
6. Criteria of treatment termination 9
7. Criteria of patient withdraw 9
8. Statistical hypothesis and sample size 10
9. Rules for dose modification 10
10. Subject selection 11
11. Clinical observation and follow-up 11
12. Trial suspension 11
13. Evaluation of efficiency and safety 11
    - 13.1 Evaluation of efficiency 11
    - 13.2 Evaluation of safety 12
    - 13.3 Genetic test 12
14. Study duration 13
15. Adverse events 13
    - 15.1 Definition 13
    - 15.2 Adverse event reporting period 13
    - 15.3 Severe adverse event (SAE) 13
    - 15.4 Report of severe adverse event 13
    - 15.5 Record and report 14
16. Statistical analysis 15
    - 16.1 Data set for statistical analysis 15
    - 16.2 Selection of statistical method 16
17. Data management 16

18. Quality control and quality assurance 17
19. Ethical Principle 17
19.1 Institutional Ethics Committee (IEC) 17
19.2 Informed consent form (ICF 18
19.3 Security measures 18
20. Data retention and summary 18
20.1 Data retention 18
20.2 Summary of trial 18

1. Study protocol summary

**Study title**	Adjuvant carboplatin and paclitaxel in triple-negative breast cancer (PATTERN): a randomized, multicenter, phase 3 trial
**Study institute**	Primary institute: Fudan University Shanghai Cancer Center<br>Participating institutes:<br>Chongqing Cancer Hospital<br>Shanghai First Maternity and Infant Hospital<br>Fudan University Obstetrics and Gynecology Hospital<br>Fujian Medical University Union Hospital<br>Shanghai Sixth People's Hospital<br>Tongji University School of Medicine Yangpu Hospital<br>The International Peace Maternity & Child Health Hospital of China Welfare Institute<br>Shanghai Ninth People's Hospital Huangpu Branch<br>Shanghai Zhongshan Hospital<br>Shanghai Huashan Hospital<br>Shanghai Tenth People's Hospital
**Study objective**	To compare the efficiency and safety between carboplatin plus paclitaxel and anthracycline followed by docetaxel when used in adjuvant chemotherapy for triple-negative breast cancer
**Study design**	Open, multi-center, prospective, randomized, controlled study
**Study population**	Patients with early-stage operable triple-negative breast cancer
**Number of subjects**	645 cases

**Treatment protocol**	adjuvant chemotherapy:<br>CEF-T group (three cycles of cyclophosphamide, epirubicin and fluorouracil followed by three cycles of docetaxel)<br>PCb group (six cycles of paclitaxel plus carboplatin)
**Evaluation parameters**	disease-free survival (DFS), distant disease-free survival (DDFS), relapse-free survival (RFS) overall survival (OS) and toxicity. In 2017, additional exploratory endpoints were amended for DFS in gBRCA1 mutation carriers and homologous recombination repair (HRR)-related gene mutation carriers.
**Evaluation for safety**	Based on physical examination, laboratory examination, adverse event, and severe adverse event. The adverse events are observed and staged based on Common Terminology Criteria for Adverse Events NCI-CTCAE 4.0.
**Statistical analysis**	All patients were included in the analysis according to the groups to which they were originally assigned (intention-to-treat analysis). All analyses were done using R programming language (http://cran.r-project.org) and STATA statistical software (StataCorp LLC).
**Expected trial duration**	8 years

2. Experiment background

In patients with early-stage triple-negative breast cancer (TNBC), although the efficacy of chemotherapy for this disease has advanced, the prognosis of these patients remains suboptimal. Both BRCA-associated breast cancer and sporadic TNBC have characteristics consistent with abnormal DNA repair and genome-wide instability, which lend support to the use of DNA-damaging compounds such as platinum. In previous investigations conducted in

neoadjuvant and advanced settings, the addition of carboplatin showed superior efficacy than standard therapy. However, the role of platinum-based adjuvant chemotherapy in TNBC patients remains controversial.

3. Experiment objective

This study intends to investigate whether a carboplatin plus paclitaxel regimen given as adjuvant treatment in TNBC would be superior to a standard regimen of anthracycline and docetaxel-based adjuvant chemotherapy.

Primary endpoint of study: disease-free survival (DFS)

Secondary endpoints of the study: distant disease-free survival (DDFS), distant disease-free survival (DDFS), relapse-free survival (RFS), overall survival (OS), toxicity, as well as DFS in gBRCA1 mutation carriers and homologous recombination repair (HRR)-related gene mutation carriers.

4. Study design

The trial was designed to determine whether the PCb was superior to the CEF-T in the adjuvant settings of TNBC. Patients were randomly assigned to receive paclitaxel 80 mg/m2 and carboplatin (area under the curve [AUC]= 2) on day 1, 8, 15 every 28 days for six cycles or fluorouracil 500 mg/m2, epirubicin 100 mg/m2, and cyclophosphamide 500 mg/m2 intravenously on day 1 every 21 days for three cycles followed by docetaxel 100 mg/m2 intravenously on day 1 every 21 days for three cycles.

Chemotherapy should be initially administrated within 8 weeks after initial breast cancer surgery. Concurrent or extended treatments of adjuvant capecitabine as well as other chemotherapeutic regimens were forbidden. Adjuvant radiotherapy was initiated within 4 weeks after the last cycle of chemotherapy if necessary. Radiation was mandatory for all patients who had undergone breast conserving surgery. Radiotherapy procedures had to be similar for both groups at a given center.

5. Participants enrollment

5.1 Inclusion criteria:

1) Age: 18-70 years old;

2) Patient has localized invasive breast carcinoma and the ER, PR, HER2 status were confirmed by histopathology after early breast cancer surgery, of which ER negative and PR negative status was less than 1% staining in the nuclei, HER2-negative was defined by local testing using the ASCO/CAP HER2 test guideline（HER2-negative breast cancer (based on most recently analyzed biopsy) defined as a negative in situ hybridization test or an Immunohistochemistry (IHC) status of 0, 1+ or 2+. If IHC is 2+, a negative in situ hybridization (Fluorescence in situ hybridization (FISH), Chromogenic in situ hybridization (CISH), or Silver in situ hybridization (SISH)) test is required by local laboratory testing.), positive lymph node or negative lymph node with tumor size > 1.0cm

3) Has an Eastern Cooperative Oncology Group (ECOG) performance status of 0 or 1;

4) No concurrent malignancy (except controlled cervical carcinoma in situ or basal cell carcinoma of skin);

5) Has adequate organ function meeting the following criteria: (1) adequate bone marrow function: hemoglobin ≥ 90 g/L (no blood transfusion within 14 days); absolute neutrophil count ≥ 1.5 x 109 /L; platelet count ≥ 100 * 109 /L; (2)adequate liver and kidney function: Alanine Aminotransferase (ALT) ≤ 3×upper limit of normal (ULN), Aspartate Aminotransferase (AST) ≤ 3×ULN, Total Bilirubin (TBIL)≤ 1.5×ULN, serum creatinine ≤ 1×ULN，and with endogenous creatinine clearance rate of >50 ml/min (Cockcroft-Gault formula).

6) No coagulation abnormality;

7) Normal heart function, with normal ECG and LVEF ≥50%;

8) Women of childbearing age agree to take reliable contraceptive measures during clinical trials, and negative serum or urine pregnancy test within 7 days prior to administration;

9) No coagulation abnormality;

10) Participants voluntarily joined the study, has signed informed consent before any trial related activities are conducted, has good compliance and has agreed to follow-up.

5.2 Exclusion criteria

1) Has received neoadjuvant therapy (include chemotherapy, targeted therapy, radiotherapy or endocrine therapy）;

2) Has bilateral breast cancer;

3) Has previous history of additional malignancy, with the exception of adequately treated basal cell carcinoma and cervical carcinoma in situ.

4) Has recurrent or metastatic (Stage 4) breast cancer;

5) Has any >T4 lesion (UICC1987) (with skin involvement, mass adhesion and fixation, and inflammatory breast cancer);

6) Is pregnant, is breast feeding women, or women of childbearing age who cannot practice effective contraceptives;

7) Patients participating in other clinical trials at the same time;

8) Has severe organ dysfunction (cardiopulmonary liver and kidney) insufficiency, left ventricular ejection fraction (LVEF) < 50% (cardiac ultrasound); severe cardio cerebral vascular disease within the 6 months previous of randomization (such as unstable angina, chronic heart failure, uncontrolled hypertension with blood pressure>150/90mmgh, myocardial infarction, or cerebral blood vessel); diabetic patients with poor blood glucose control; patients with severe hypertension;

9) Has known pre-existing neuropathy, or allergy to cremophor-containing medications;

10) Has severe or uncontrolled infection;

6. Criteria of treatment termination

1) The medical condition that is harmful to the health of the patient, the researcher believes that it should be discontinued

2) Severe adverse event occurs thus unsuitable to accept further treatment;

3) The patient was still unable to receive the prescribed treatment within 42 days after the last dose of the last cycle.

4) Disease recurrence

5) Superior administrative agency forces trial suspension due to certain reason;

7. Criteria of patient withdraw

1) Requirement to withdraw the consent by subjects

Subjects are free to withdraw from the trial at any time according to his/her own intention. In case subjects don’t return for follow-up as schedule, they should be contacted by any possible means. The results of subjects should be recorded to the best. For subjects withdrawn from trial, reason for withdrawal should be inquired, as well as access to final clinical follow-up and unrelieved adverse events. If patient withdraws from the study and retracts the consent, and refused to disclose further information, then no further evaluation or collection for additional data is needed. Patients with interrupt of follow-up longer than 6 months should be considered as withdrawn from trial. Refer to study exit/withdrawal procedure for the data should be collected at withdrawal. The adverse event experienced by subjects as well as concurrent medication should be followed every 3 months for 2 years.

2) Researchers believe subjects should exit trial due to medical-related consideration

For the purpose of protecting the subjects, they should be required to exit trial even before completion of scheduled follow-ups; Use of other therapy or medication contraindicated to combined usage that affects the evaluation of safety; Severe adverse event occurs thus unsuitable to accept further clinical visits; Subjects fail to visit on time or lost, featured with poor compliance.

8. Statistical hypothesis and sample size

The trial was designed to determine whether the PCb was superior to the CEF-T in the adjuvant settings of TNBC. A two-sided log-rank test with an overall sample size of 614 participants (307 in the CEF-T group and 307 in the PCb group) achieved 80.0% power at a 0.05 significance level, to detect a HR of 0.60 when the proportion survival in the CEF-T group was 82.0% and 89.0% in the PCb group, with 112 DFS events expected (69 in control group and 43 in test group) after 5-year follow-up time. According 5% loss to follow-up, **645** randomized patients were required. No interim analysis was planned for DFS.

9. Rules for dose modification

Dose adjustments were made as follows: if, on the planned day of therapy, absolute neutrophil count (ANC) was less than 1.5 X 10^9/L and/or platelet count was less than 100 X 10^9/L, chemotherapy was delayed until the criteria were met. A maximum postponement of 3 weeks was allowed. In both treatment arms, dose reductions were performed in patients who had grade 4 neutropenia lasting more than 7 days or in patients with febrile neutropenia (≥ 38.5°C). Treatment was interrupted and the dose was reduced in patients who experienced a second occurrence of a given grade 2 nonhematologic toxicity (except alopecia, nausea/vomiting, and peripheral neuropathy) or any grade 3 toxicity. Furthermore, all nonhematologic toxicities ((except alopecia and nausea/vomiting) should have subsided to grade 1 or less before re-treatment. For peripheral neuropathy, docetaxel was reduced for grade 2 and was discontinued for grade 3. The following dose modifications schemes were used: All drugs reduced by 25% at the first time and by 50% at the second time. No more than two reductions per patient.

10. Subject selection

The medical history of subjects is thoroughly reviewed and complete physical examination at baseline including blood routine examination, liver and kidney function test, ECG, cardiac ultrasound, chest CT, breast and abdominal ultrasound, bone scan (if necessary), is performed to all subjects.

11. Clinical observation and follow-up

Before start of every cycle of adjuvant chemotherapy, physical examination is performed. The tolerability of chemotherapy was evaluated before each cycle. In addition, an electrocardiograph and an absolute blood count were performed on day 21, and nonhematologic toxicity was evaluated during the period between cycles. Toxicity was graded according to National Cancer Institute Common Toxicity Criteria version 4.0 (updated in 2010).

All patients underwent routine follow-up after the completion of adjuvant chemotherapy. For the first 2 years after surgery, the tumor marker, CA15-3, CEA and AFP, are tested every 3 months, then the follow up is done every 6 months. For the first 2 years, abdominal ultrasound is performed every 6 months, and then the follow up is done once a year. Chest CT and mammography are performed once a year.

12. Trial suspension

Trial suspension refers to the condition that all tests are terminated prior to the end of clinical trial schedule due to following reasons, with the major purpose of protecting the interest of subjects and maintaining study quality, and avoid unnecessary financial losses.

1) Occurrence of unacceptable adverse events;
2) Researchers believe necessary to terminate the trial;
3) Subjects voluntarily require to exit;
4) Severe violation of study protocol.

13. Evaluation of efficiency and safety

13.1 Evaluation of efficiency

The primary endpoint was disease-free survival (DFS), defined as time from random assignment to first relapse (local, regional and distant), contralateral breast cancer, second primary cancer (other than squamous or basal cell carcinoma of the skin, melanoma in situ, or carcinoma in situ), or death with any cause. Secondary end points included distant disease-free survival (DDFS), defined as time from random assignment to distant recurrence or death; relapse-free survival (RFS), defined as the time from the date of randomization to local, regional, distant relapse or death; overall survival (OS), defined as time from randomization until death with any cause; and toxicity.

13.2 Evaluation of safety

Evaluation of safety includes but not limits to observation and report of all adverse events and severe adverse events, laboratory tests, physical examination, physical status rating scale and electrocardiogram.

The adverse events are observed and staged based on Common Terminology Criteria for Adverse Events NCI-CTCAE 4.0. Investigators are responsible to take therapeutic measures according to adverse events. Investigators should fill in the report in the corresponding form of case report and validate whether the adverse event or severe adverse event relates with this study.

13.3 Genetic test

In initial trial design, we hypothesized that carriers with deleterious gBRCA1/2 mutation might get more survival benefits from carboplatin-containing chemotherapy compared with the traditional regimen. For all the participants, germline mutation test of BRCA1/2 gene was recommended but not mandatory. BRCA1/2 mutations were determined from blood samples obtained before chemotherapy. Since the results of BRCA test need time and we could not get mutation information before chemotherapy, randomisation could not be stratified according to the gBRCA1/2 status. All coding regions and exon-intron boundaries of the BRCA1/2 genes were screened. Genetic test was conducted in the center laboratory of Fudan University Shanghai Cancer Center13. All mutations considered pathogenic or likely-pathogenic were

validated via Sanger sequencing. In 2017, the protocol was amended, we further tested a set of 12 genes involved in homologous recombination repair (HRR)——ATM, ATR, BARD1, BRCA1, BRCA2, BRIP1, CHEK2, FANCM, PALB2, RAD51C, RAD51D, and RECQL——for germline mutation. The DFS in patients with or without mutation was evaluated..

14. Study duration

8 years (containing 5-year follow-up)

15. Adverse events

15.1 Definition

An adverse event (AE) is any undesirable medical event experienced by clinical trial subject after certain examination, administration of test compound or performance of certain therapy; however, AE does not always reflect a causal relationship with intervention.

15.2 Adverse event reporting period

The reporting period starts from enrollment and lasts till the final follow up. Any adverse event happens during this period should be filled in the case report form.

15.3 Severe adverse event (SAE)

An adverse event is defined as severe adverse event if it agrees with one or more following criteria:

1) Death;
2) Life-threatening；
3) Hospitalization (initial or prolonged)
4) Disability or Permanent Damage
5) Congenital Anomaly/Birth Defect, important Medical Events

15.4 Report of severe adverse event

For all severe adverse events the trial should be suspended immediately and corresponding measures to protect subjects should be practiced. Severe adverse events will be recorded in a table and reported to the director of the unit and the sponsor within 24 hours by phone or fax. The investigator shall fill in the severe adverse event report form and fax it to State Food and Drug Administration. Investigator should follow the severe adverse event till resolution. Relevant medical documents should be achieved within original material, including report sheet of laboratory test (e.g. X-ray examination, electrocardiogram, etc).

15.5 Record and report

For adverse event occurred during the trial, its time of onset, symptoms, severity, duration, treatment and prognosis should be recorded in the case report, so as to evaluate its significance with test compound. The detailed record should be provided, signed and dated by investigators. The adverse events will be graded based on NCI-CTC 4.0. For each symptom, the highest grade experienced since last follow-up should be reported.

Determination of relationship between adverse events and clinical trial:

Attribute to one of the five categories: definitely related, probably related, probably unrelated, unrelated and not appreciable. The first two categories are considered as adverse event and the proportions of adverse events will be calculated.

Definite attribution: The chronological order of adverse event after trial onset is reasonable and the reaction is consistent with the known type of reaction.

Probably related: The chronological order of adverse event after trial onset is reasonable and the reaction is consistent with the known type of reaction; the clinical condition or alternative intervention may also cause such reaction.

Probably unrelated: The chronological order of adverse event after trial onset is less reasonable and the reaction is less consistent with the known type of reaction; the clinical condition or alternative intervention may also cause such

reaction.

Unrelated: The chronological order of adverse event after trial onset is unreasonable and the reaction is inconsistent with the known type of reaction; the clinical condition or alternative intervention may also cause such reaction. Improvement of disease status or stop intervention other than test compounds lead to elimination of such reaction, which will relapse with the restart of alternative intervention.

Not appreciable: The onset of reaction and trial lack clear chronological order and the reaction is similar with known reaction type. Concurrent use of other medication may cause same reaction.

16. Statistical analysis

The statistical analysis will be accomplished by professional statistical workers, who will be involved through the whole process from study design, practice to analysis and summary. Statistical analysis plan will be made after trial protocol and completion of case report form, and the statistical analysis report should be submitted based on necessary modification to data analysis.

16.1 Data set for statistical analysis

1) Full Analysis Set (FAS):

Based on the principle of intention-to-treat (ITT), full analysis set (FAS) data set is defined as follows: For all subjects enrolled and finish at least one follow-up, all their case materials, including those unable to fulfill all visits, will be included in the final results with the data observed at the last time point. FAS data set will be used in dropout analysis, balance analysis of the baseline parameters, analysis of major parameters, and analysis and safety parameter. For the missing data resulted from drop-outs, the unfinished points of visits will be carried forward with the last observation result; the intermediate missing values of cases completed the study will be carried forward with the data mostly recent to the missing points. If no data can be used to carry forward, then the data will be treated as missing data; if subject get lost after enrollment with no appreciable data achieved, then there is no need to do carry forward but treat

the relevant data as missing data.

2) Per-Protocol population (PP) data set:

The PP data set is defined restricted to all the cases reports from subjects adhered to trial protocol, with good compliance and fulfilled required contents in case report form, as well as the observation record documents from subjects whose compliance satisfies the study protocol requirement. The statistical analysis for efficiency in this study will be based on PP data set.

16.2 Selection of statistical method

1) All analyses were done using R programming language and environment version (http://cran.r-project.org) and STATA statistical software (StataCorp LLC).

2) Two-sided *P* values will be calculated for all statistical tests. A value of P <0.05 is considered significant.

3) For continuous and categorical factors, the Wilcoxon rank sum test and the chi-square test (or Fisher exact test when necessary), were used to evaluate the difference between the two groups.

4) The Cox method to calculate HRs and 95% CI for the between-group comparisons of outcomes and the log-rank test to calculate corresponding P values.

17. Data management

a) For all patients filled in the informed consent and eligible for screening, all items included in the case report form should be filled carefully and in detail. No blank or omission is allowed (for item with no records, please draw a hyphen);

b) All data in the case report form should be checked with medical records of subject to avoid any mistake;

c) The case reports are used as raw data. In case any correction is needed, the corrected data should be indicated with cross line in the margin and signed and dated by investigator;

d) The copy of laboratory result sheet should be paste on the specific place of case report form;

e) Significantly higher data or those fall outside the clinically acceptable range should be confirmed and supplemented with necessary explanation by investigators;

Please refer to instruction for filling in the case report form.

18. Quality control and quality assurance

Unified study protocol, standard operating procedures and quality control procedures should be established by all participating hospitals of this trial. Regular supervision and inspection will be performed during the trial to ensure the implementation of study protocol. The raw data will be reviewed to ensure the consistency with data in the case report form.

19. Ethical Principle

The study procedure must strictly conform to the requirement of *Good Clinical Practice* of SFDA and the *Declaration of Helsinki*.

19.1 Institutional Ethics Committee (IEC)

This protocol and written informed consent as well as material directly related with subjects shall be submitted to ethics committee of the Fudan University Shanghai Cancer Center. The trial can only initiate after achievement of written approval of the ethics committee. Investigator are required to submit annual report (if applicable) to the ethics committee at least once a year. When the study is suspended and/or completed, the ethics committee should be informed by investigators with written notification; the ethics committee should be informed timely about any change during the study process (e.g. the modification to protocol and/or informed consent form), and the change can only be realized after approval of ethics committee, unless those made to eliminate the obvious and immediate risk to subjects. The ethics committee should be informed anyway under above-mentioned circumstance.

For the participant centers, independent institutional review board of the participating hospitals approved the study protocol separately.

19.2 Informed consent form (ICF)

Prior to enrollment the investigators are responsible for oral and written consent about information including objective, procedure and potential risks of the study to every subject. The subject should be informed about the right to decide whether to participate in the trial and that subject is free to withdraw from trial any time willingly. Subjects or their legal representatives will read and understand the informed consent form and sign it, and keep the copy of signature page.

19.3 Security measures

The medical records (medical history, physical and chemical tests report, etc.) will be integrally stored by hospitals. The doctors (investigators), professional academic committee, ethics committee and health supervision departments will be allowed with access to medical records. The result of this study may be submitted for publishing on medical journals. However, any open report concerning this study will not disclosure individual identity of patients. The privacy of personal medical information of patients should be protected by all permissible means.

20. Data retention and summary

20.1 Data retention

The case report forms will be confirmed with signature by investigators. After completion of trial, all case report forms, detailed materials about classic cases and clinical trial record forms will be sent to sponsor. Original materials relevant to subjects, laboratory results, signed informed consent originals and copy of case report forms will be kept by investigators.

20.2 Summary of trial

The fulfillment of “summary report of clinical trial” will be the responsibility of study sponsor.

Participating centers

	Hospital
1	Fudan University Shanghai Cancer Center
2	Chongqing Cancer Hospital
3	Shanghai First Maternity and Infant Hospital
4	Fudan University Obstetrics and Gynecology Hospital
5	Fujian Medical University Union Hospital
6	Shanghai Sixth People’s Hospital
7	Tongji University School of Medicine Yangpu Hospital
8	The International Peace Maternity & Child Health Hospital of China Welfare Institute
9	Shanghai Ninth People’s Hospital Huangpu Branch
10	Shanghai Zhongshan Hospital
11	Shanghai Huashan Hospital
12	Shanghai Tenth People’s Hospital

Enrollment method of patients: competitive
