# Supplementary material for: Effect of Adjuvant Paclitaxel and Carboplatin on Survival in Women With Triple-Negative Breast Cancer: A Phase 3 Randomized Clinical Trial
Source: JAMA Oncol. 2020 Aug 13;6(9):1–8. doi: 10.1001/jamaoncol.2020.2965 (PMC7426881; doi:10.1001/jamaoncol.2020.2965)
Supplement: Supplement 2. — eMethods. Protocol and Participating Hospitals eTable 1. Recruitment of Patients by Hospital and Institution eTable 2. Genes with Germline Mutation in 405 TNBC Using Whole Exome Sequencing Assay eTable 3. Grade 3 to 4 Treatment-Related Adverse Events eFigure 1. Kaplan-Meier Plots of (A) Distant Disease-Free Survival and (B) Relapse-Free Survival eFigure 2. Kaplan-Meier Plots of Disease-Free Survival According to BRCA1 Germline Mutation (A) and HRR Germline Mutation Status (B) [file jamaoncol-e202965-s002.pdf]

Supplementary Online Content

Yu K-D, Ye F-G, He M, et al. Effect of adjuvant carboplatin and paclitaxel on survival in women with triple-negative breast cancer: a randomized phase 3 clinical trial. *JAMA Oncol*. Published online August 13, 2020.
do:10.1001/jamaoncol.2020.2965

eMethods. Protocol and Participating Hospitals
eTable 1. Recruitment of Patients by Hospital and Institution
eTable 2. Genes with Germline Mutation in 405 TNBC Using Whole Exome Sequencing Assay
eTable 3. Grade 3 to 4 Treatment-Related Adverse Events
eFigure 1. Kaplan-Meier Plots of (A) Distant Disease-Free Survival and (B) Relapse-Free Survival
eFigure 2. Kaplan-Meier Plots of Disease-Free Survival According to BRCA1 Germline Mutation (A) and HRR Germline Mutation Status (B)

This supplementary material has been provided by the authors to give readers additional information about their work.

© 2020 Yu K-D et al. *JAMA Oncology.*

eMethods. Protocol and Participating Hospitals

The protocol was provided online at http://www.sibcs.com/page165. The original number of participating hospitals were 12, and the actual recruitment was conducted in 9 hospitals (**eTable 1**), including Fudan University Shanghai Cancer Center, Chongqing Cancer Hospital, Shanghai First Maternity and Infant Hospital, Fudan University Obstetrics and Gynecology Hospital, Fujian Medical University Union Hospital, Shanghai Sixth People's Hospital, Tongji University School of Medicine Yangpu Hospital, The International Peace Maternity & Child Health, Hospital of China Welfare Institute, and Shanghai Ninth People's Hospital Huangpu Branch. The enrolment of patients was competitive among the participating hospitals.

Adjuvant radiotherapy, if necessary, was initiated within 4 weeks after the last cycle of chemotherapy. Radiation was mandatory for all patients who had undergone breast-conserving surgery, and the radiotherapy procedures were similar for both groups at a given centre.

Follow-up visits were scheduled every 3 months for the first 2 years and then every 6 months for the next 3 years. Imaging examinations (mammography, computerised tomography scan, and bone scan) were performed 1 year after the initial surgery and then annually thereafter. Adverse events were recorded at each treatment visit and at each follow-up visit.

BRCA1/2 testing

All coding regions and exon-intron boundaries of the BRCA1/2 genes were screened. The average intronic sequence length was 70 bp (ranging from 5 to 204 bp). Large genomic rearrangements in BRCA1/2 were not included in the present study. The sequencing results were compared with the BRCA1 (NM_007294.3) and BRCA2 (NM_000059.3) reference sequences for variant detection. All mutations considered disease-associated (pathogenic or likely pathogenic) were validated via Sanger sequencing.

Genomic DNA was isolated from peripheral lymphocytes using a TGuide M16 automatic extraction machine (Tiangen Biotechnology, Beijing, China). The DNA concentration was quantified using a NanoDrop ND2000 (NanoDrop Technologies, Wilmington, DE, USA) spectrophotometer, and the samples were diluted to 20-50 ng/μl if the DNA concentration was higher than 50 ng/μl. All DNA samples were amplified in two separate multiplex PCR assays. Self-designed primers were used. Each

© 2020 Yu K-D et al. *JAMA Oncology.*

amplification reaction was prepared by mixing 3 µl of the genomic DNA, 8 µl of each primer panel, 12.5 µl of the KAPA2G Robust hot start ready mix (Kapa Biosystems, Wilmington, MA, USA) and 1.5 µl of H2O. The PCR program was 95°C for 4 min followed by 18 cycles of 98°C for 15 s and 60°C for 4 min. The PCR products were cleaned up using AMPure XP Beads (Beckman Coulter, Pasadena, CA, USA).

Barcoding was performed in a 20-µl reaction mixture that contained 8 µl of the cleaned PCR products, 10 µl of KAPA2G Robust hot start ready mix (Kapa Biosystems, Pasadena, CA, USA), 1 µmol/L barcode F primers and 1 µmol/L barcode R primer. The reaction was performed in a conventional PCR thermal cycler. The barcoded PCR products from the various samples were cleaned up using AMPure XP Beads (Beckman Coulter, Pasadena, CA, USA). The purified PCR product library was quantified using a Qubit Fluorometer (Thermo Fisher Scientific, Waltham, MA, USA). Based on library quantitation, the PCR products were pooled together in equal molar ratios. The purified libraries were routinely sequenced on a NextSeq 500 sequencer (Illumina, San Diego, CA, USA) using the 2x150 bp end sequencing protocol.

Analysis of sequencing data

Demultiplexed, compressed FASTQ files were generated from BCL using bcl2fastq Conversion Software v1.8.4 (Illumina, San Diego, CA, USA). For all successful sequencing runs, the read depth was 30x at any given position, with 100x mean coverage across the entire targeted sequence and Q30 at greater than 75% of reads. The variant calling and coverage of each captured region were analyzed using an in-house-developed bioinformatics pipeline based on the general analysis algorithm pipeline. Briefly, the reads were mapped to the hg19 version of the human reference genome (GRCh37) and then filtered to remove off-target and poor-quality reads. Variants were identified and annotated. The variants and annotation results were transferred into Excel spreadsheets. The mutations were classified as benign, likely-benign, variants of uncertain significance, likely-pathogenic, and pathogenic. If applicable, detailed information was obtained using the gene-specific databases dbSNP, ClinVar, and BIC. Subsequently, a manual literature search was performed using a Google search in PubMed, Science-Direct, and BioMed Central to confirm that there had been no previous reports on each specific mutation. Novel mutations were defined when there was no match to the reference single-nucleotide polymorphism RS numbers in the dbSNP database. Mutations were interpreted as positive for a

© 2020 Yu K-D et al. *JAMA Oncology.*

pathogenic mutation when (1) nonsense mutations prematurely terminated the protein product of BRCA1 at least 10 amino acids from the C-terminus or the protein product of BRCA2 at least 110 amino acids from the C-terminus; (2) frameshift insertions or deletions resulted in the expression of an abnormal or truncated protein product; (3) mutations in noncoding intervening sequence at splicing sites caused abnormal processing of the mRNA transcript; or (4) missense mutations and non-frameshift insertions or deletions were defined as pathogenic in a database and/or published study. The mutations with clear pathogenic impacts reported in previous studies were selected for further analysis. A subset of variants, including known variants that were pathogenic or likely pathogenic and newly identified variants with functional damage, was confirmed by conventional Sanger sequencing using the BigDye Terminator v3.1 Cycle Sequencing Kit (Thermo Fisher Scientific, Waltham, MA, USA). Variants that could not be confirmed were excluded from further analysis.

Multigene testing

The multigene panel includes 12 breast cancer homologous recombination repair (HRR) associated susceptibility genes (**eTable 2**). They were ATM, ATR, BARD1, BRCA1, BRCA2, BRIP1, CHEK2, FANCM, PALB2, RAD51C, RAD51D, and RECQL. All coding regions and exon-intron boundaries of the genes were screened. The multiplex PCR, barcoding and Illumina sequencing, and analysis of sequencing data were conducted following aforementioned procedures. A subset of variants, including known variants that were pathogenic or likely pathogenic and newly identified variants with functional damage, was confirmed by conventional Sanger sequencing using the BigDye Terminator v3.1 Cycle Sequencing Kit (Thermo Fisher Scientific, Waltham, MA, USA). Variants that could not be confirmed were excluded from further analysis.

Outcomes

The primary endpoint was disease-free survival (DFS). Events of DFS included non-invasive and invasive breast cancer recurrences (local, regional, or distant), second primary non-invasive and invasive breast and other cancers other than basal/squamous-cell carcinoma of the skin and carcinoma in situ of the cervix, and death from any cause. Secondary endpoints included the following: distant disease-free survival (DDFS), defined as the time from random assignment to distant recurrence or death; relapse-free survival (RFS), defined as the time from the date of

© 2020 Yu K-D et al. *JAMA Oncology.*

randomisation to local, regional, distant relapse or death whichever occurred first; overall survival (OS), defined as the time from randomisation to death with any cause; and toxicity. Another prespecified secondary endpoint was DFS in gBRCA1 mutation carriers. An exploratory analysis of the interaction between carboplatin-containing chemotherapy and HRR-related gene mutation was amended and added in 2017.

© 2020 Yu K-D et al. *JAMA Oncology*.

eTables

eTable 1. Recruitment of Patients by Hospital and Institution

Hospital	N=647
Fudan University Shanghai Cancer Center	521
Chongqing Cancer Hospital	36
Shanghai First Maternity and Infant Hospital	21
Fudan University Obstetrics and Gynecology Hospital	20
Fujian Medical University Union Hospital	11
Shanghai Sixth People’s Hospital	10
Tongji University School of Medicine Yangpu Hospital	10
The International Peace Maternity & Child Health Hospital of China Welfare Institute	10
Shanghai Ninth People’s Hospital Huangpu Branch	8

© 2020 Yu K-D et al. *JAMA Oncology.*

eTable 2. Genes with germline mutation in 405 TNBC using whole exome sequencing assay

Gene	Number of cases with deleterious mutation	Frequency (%)	Putative HRR gene
BRCA1	33	8.1	Yes
BRCA2	15	3.7	Yes
RAD51D	13	3.2	Yes
PALB2	5	1.2	Yes
RAD51C	5	1.2	Yes
BRIP1	4	1.0	Yes
FANCM	2	0.5	Yes
GJB2	2	0.5	No
ATM	1	0.2	Yes
ATR	1	0.2	Yes
BARD1	1	0.2	Yes
CHEK2	1	0.2	Yes
MAP2K1	1	0.2	No
MSH6	1	0.2	No
MUTYH	1	0.2	No
PDGFRA	1	0.2	No
PTCH1	1	0.2	No
PTEN	1	0.2	No
RECQL	1	0.2	Yes
SETBP1	1	0.2	No
SUFU	1	0.2	No
TP53	1	0.2	No
All	93	23.0	

© 2020 Yu K-D et al. *JAMA Oncology.*

eTable 3. Grade 3 to 4 Treatment-Related Adverse Events

Adverse events	CEF-T (n=320)	PCb (n=322)	P
Haematologic toxic effects			
Neutropaenia	297 (92.8%)	283 (87.9%)	0.035
Febrile neutropaenia	30 (9.4%)	3 (0.9%)	<0.001
Leukopenia	288 (90.0%)	273 (84.8%)	0.047
Anaemia	5 (1.6%)	32 (9.9%)	<0.001
Thrombocytopaenia	5 (1.6%)	16 (5.0%)	0.015
Non-haematologic toxic effects			
Infections	6 (1.9%)	5 (1.6%)	0.75
Nausea	3 (0.9%)	1 (0.3%)	0.31
Vomiting	6 (1.9%)	3 (0.9%)	0.31
Diarrhea	8 (2.5%)	3 (0.9%)	0.13
Abdominal pain	5 (1.6%)	2 (0.6%)	0.25
Constipation	3 (0.9%)	0	0.08
Peripheral sensory neuropathy	3 (0.9%)	12 (3.7%)	0.02
Edema	10 (3.1%)	2 (0.6%)	0.02
Arthralgia/myalgia	4 (1.3%)	1 (0.3%)	0.18
Fatigue	3 (0.9%)	3 (0.9%)	0.99
Epistaxis	2 (0.6%)	0	0.16
Laboratory-assessed items			
Alanine aminotransferase increased	6 (1.9%)	7 (2.1%)	0.79
Aspartate aminotransferase increased	5 (1.6%)	6 (1.9%)	0.77

© 2020 Yu K-D et al. *JAMA Oncology.*

eFigures

eFigure 1: Kaplan-Meier Plots of (A) Distant Disease-Free Survival and (B) Relapse-Free Survival.

eFigure 1

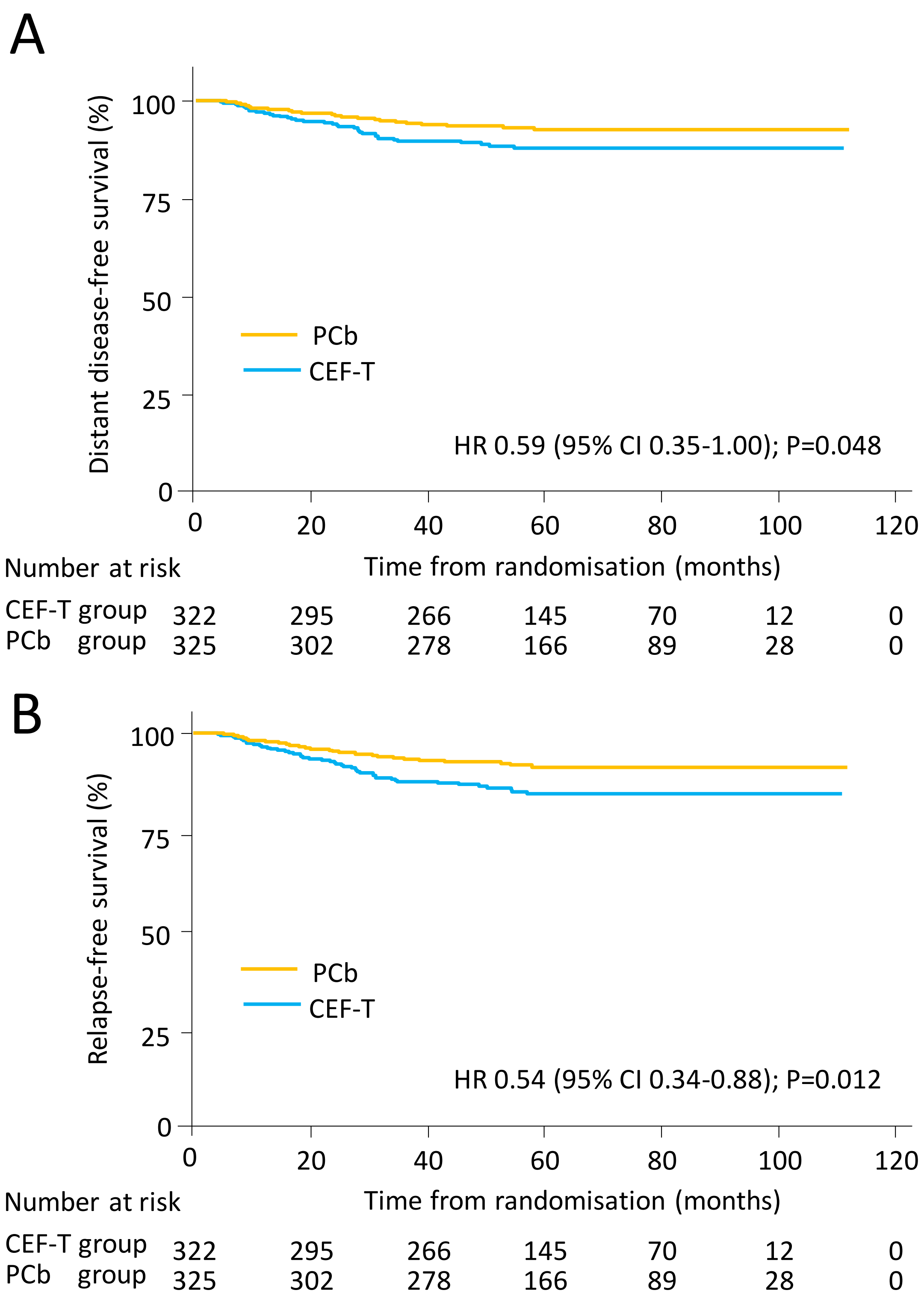

© 2020 Yu K-D et al. *JAMA Oncology.*

eFigure 2: Kaplan-Meier Plots of Disease-Free Survival According to BRCA1 Germline Mutation (A) and HRR Germline Mutation Status (B)

eFigure 2

A

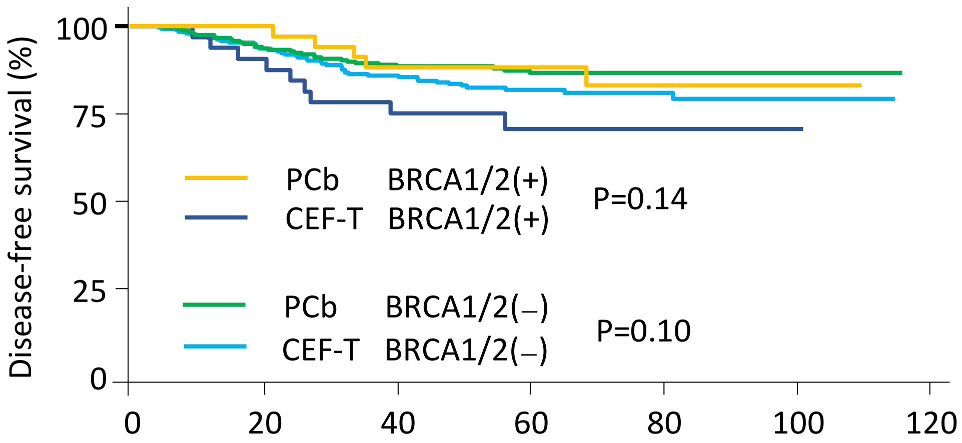

Number at risk

		0	20	40	60	80	100	120
PCb	BRCA1/2(+)	34	34	30	22	10	2	0
CEF-T	BRCA1/2(+)	32	28	24	15	8	2	0
PCb	BRCA1/2(–)	235	218	202	118	65	21	0
CEF-T	BRCA1/2(–)	237	218	198	109	44	10	0

B

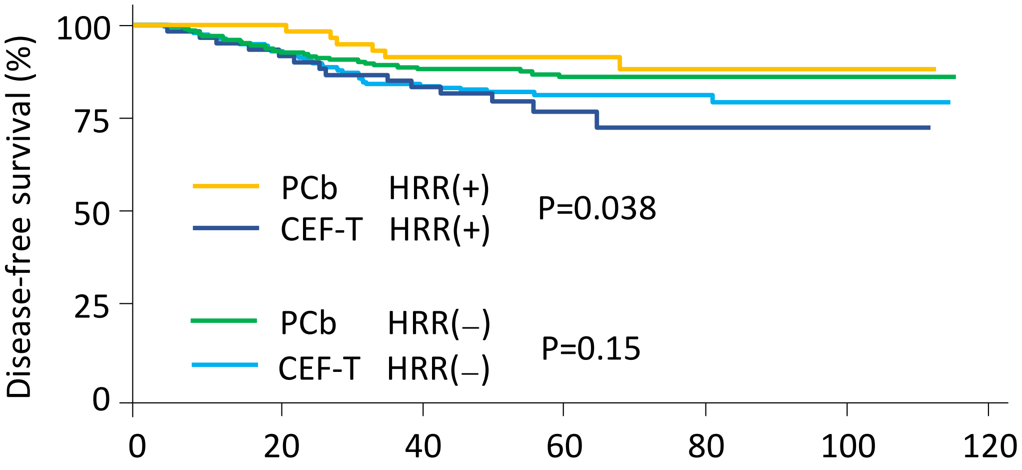

Number at risk

		0	20	40	60	80	100	120
PCb	HRR(+)	59	58	53	35	20	8	0
CEF-T	HRR(+)	61	55	50	23	8	2	0
PCb	HRR(–)	202	187	173	100	53	15	0
CEF-T	HRR(–)	199	182	163	93	41	8	0

© 2020 Yu K-D et al. *JAMA Oncology.*
