# Supplementary material for: Effect of Adjuvant Paclitaxel and Carboplatin on Survival in Women With Triple-Negative Breast Cancer: A Phase 3 Randomized Clinical Trial
Source: JAMA Oncol. 2020 Aug 13;6(9):1–8. doi: 10.1001/jamaoncol.2020.2965 (PMC7426881; doi:10.1001/jamaoncol.2020.2965)
Supplement: Supplement 3. — Data Sharing Statement [file jamaoncol-e202965-s003.pdf]

Data Sharing Statement

Yu. Effect of Adjuvant Paclitaxel and Carboplatin on Survival in Women With Triple-Negative Breast Cancer. *JAMA Oncol*. Published August 13, 2020. 10.1001/jamaoncol.2020.2965

Data
Data available: Yes
Data types: Deidentified participant data, Other (please specify)
Additional Information: Without genetic information such as BRCA1/2 status
How to access data: Individual participant data that underlie the results reported in this article expect germline mutation information, after de-identification (text, tables, figures, and appendices). Proposals should be directed to yukeda@163.com. To gain access, data requestors will need to sign a data access agreement.
When available: With publication

Supporting Documents
Document types: None

Additional Information
Who can access the data: Researchers who provide a methodologically sound proposal. Especially welcome to EBCTCG.
Types of analyses: For individual participant data meta-analysis
Mechanisms of data availability: Proposals should be directed to yukeda@163.com. To gain access, data requestors will need to sign a data access agreement.
Any additional restrictions: No
